# The Anti-Inflammatory Effects of the Small Molecule Pifithrin-µ on BV2 Microglia

**DOI:** 10.1159/000370031

**Published:** 2015-02-25

**Authors:** Bobbi Fleiss, Vibol Chhor, Nazurah Rajudin, Sophie Lebon, Henrik Hagberg, Pierre Gressens, Claire Thornton

**Affiliations:** ^a^Department of Perinatal Imaging and Health, Division of Imaging Sciences and Biomedical Engineering, King's College London, King's Health Partners, St. Thomas' Hospital, London, UK; ^b^INSERM, U1141, Paris, France; ^c^University Paris Diderot, Sorbonne Paris Cité, UMRS 1141, Paris, France; ^d^PremUP, Paris, France; ^e^Department of Clinical Sciences, Sahlgrenska Academy/East Hospital, Gothenburg University, Gothenburg, Sweden

**Keywords:** Neuroinflammation, Phenotype, Neuroprotective, 2-phenylethynesulfonamide, Pifithrin-µ

## Abstract

Neonatal encephalopathy (NE) is a leading cause of childhood death and disability in term infants. Treatment options for perinatal brain injury are limited and developing therapies that target multiple pathways within the pathophysiology of NE are of great interest. Pifithrin-µ (PFT-µ) is a drug with striking neuroprotective abilities in a preclinical model of hypoxia-ischemia (HI)-induced NE wherein cell death is a substantial cause of injury. Work from neurons and tumor cells reports that PFT-µ is able to inhibit p53 binding to the mitochondria, heat shock protein (HSP)-70 substrate binding and activation of the NF-kB pathway. The purpose of this study is to understand whether the neuroprotective effects of PFT-µ also include direct effects on microglia. We utilized the microglial cell line, BV2, and we studied the dose-dependent effect of PFT-µ on M1-like and M2-like phenotype using qRT-PCR and Western blotting, including the requirement for the presence of p53 or HSP-70 in these effects. We also assessed phagocytosis and the effects of PFT-µ on genes within metabolic pathways related to phenotype. We noted that PFT-µ robustly reduced the M1-like (lipopolysaccharide, LPS-induced) BV2 response, spared the LPS-induced phagocytic ability of BV2 and had no effect on the genes related to metabolism and that effects on phenotype were partially dependent on the presence of HSP-70 but not p53. This study demonstrates that the neuroprotective effects of PFT-µ in HI-induced NE may include an anti-inflammatory effect on microglia and adds to the evidence that this drug might be of clinical interest for the treatment of NE.

## Introduction

Neonatal Encephalopathy (NE) is a leading cause of childhood death and disability in term infants [1]. Treatment options for perinatal brain injury are limited to hypothermia, which can only be applied safely to term infants diagnosed with hypoxic-ischemic encephalopathy (HIE) [2]. Unfortunately, hypothermia is only able to improve outcome for 1 out of every 7 infants treated, but critically provides proof-of-concept for treatment after injury [3]. Adjunct and alternative therapies are therefore a health care priority and, to date, those that target multiple pathways within the pathophysiology of NE [4] (such as hypothermia) have had the greatest preclinical and phase I/II success, i.e melatonin, dexmedetomidine and xenon [5,6,7,8,9]. Cell death is an important pathological process in NE and, in addition, inflammation is a chief pathophysiological process. Inflammation is mediated by microglia within the brain, and the presence and persistence of inflammation in infants with NE associates with poor long-term outcome [10,11]. As such, drugs with dual anti-cell-death anti-inflammatory properties are of great therapeutic interest.

A drug with striking neuroprotective abilities in a preclinical model of hypoxia-ischemia (HI)-induced NE [12] is a small molecule known as pifithrin-µ (PFT-µ; also known as 2-phenylethynesulfonamide, PES, or 2-phenylacetylenesulfonamide, PAS [13,14,15]). In this preclinical model, even with a 6-hour delay in administration, PFT-µ has been shown to prevent neuronal loss and white matter damage and ameliorate behavioral deficits up to 10 weeks after injury [12]. PFT-µ inhibits the association between p53 and the antiapoptotic proteins Bcl-xL and Bcl-2 at the mitochondrial surface without altering the nuclear translocation of p53 [13]. One possibility is that PFT-µ inhibits p53-mediated mitochondrial impairment also in perinatal brain injury [16,17]. PFT-µ also inhibits the interaction of the molecular chaperone heat shock protein (HSP)-70 with its many substrates [15] and effectively reduces the activation of the NF-kB pathway [18]. It is unknown whether the ability of PFT-µ to protect the developing brain from HI injury is based only on disrupting p53-dependent neuronal death, or whether this drug also directly influences microglial function via its non-p53-dependent mechanisms. Understanding any direct effects on microglia and the mechanism of any effect is the purpose of this study.

Microglia are implicated in the neuropathology of NE in term and preterm infants as well as most acute and chronic neurodegenerative diseases [for reviews, see [19,20]]. In addition, they play a crucial role in the repair and regeneration of the brain [21,22]. To participate in this diverse range of activities, microglia respond to extracellular cues to acquire distinct phenotypes. A phenotype is characterized by measuring the expression of soluble factors, cell surface markers and enzymes responsible for activities such as bactericidal and extracellular matrix remodeling and extrapolating microglial function based on our knowledge of the roles of these markers [23,24]. Markers characterizing the basal state of microglia in culture as well as the M1-like proinflammatory phenotype induced by lipopolysaccharide (LPS) and the M2-like reparatory and regeneration-supporting phenotype induced by IL-4 from microglia in vitro have been thoroughly described [25,26,27]. These markers provide a useful guide for understanding the effects of drugs on microglia in vivo as a screening tool.

We utilized the microglial cell line, BV2, which has expression and an LPS-dependent response of p53, NF-kB and HSP-70 comparable with that seen in primary murine microglia [28]. We studied the dose-dependent effect of PFT-µ on the M1-like proinflammatory and M2-like regenerative phenotype, including the requirement for the presence of p53 or HSP-70 in these effects. We noted that PFT-µ robustly reduced the M1-like (LPS-induced) BV2 response and spared the LPS-induced phagocytic ability of BV2 and that these effects were partially dependent on the presence of HSP-70 but not p53.

## Methods

### Drugs

PFT-µ (P0122; Sigma, Lyon, France) was diluted in 100% DMSO to 5 mg/ml and then in PBS to a stock concentration of 10 µM. LPS (L2880, lot 050M4014; Sigma) was diluted in PBS to a stock concentration of 0.1 mg/ml. Interleukin-4 (IL-4) was from R&D systems (Lille Cedex, France), diluted in PBS and 0.1% bovine serum albumin to create a stock solution of 2 µg/ml.

### Microglial BV2 Culture

The immortalized microglia cell line, BV2, was donated by Professor R. Donato (University of Perugia, Perugia, Italy); it was generated by infecting primary mouse microglia cultures with a v-raf/v-myc oncogene-carrying retrovirus (J2) [29]. BV2 were cultured, as previously reported, in RPMI media containing gentamycin and supplemented with 10% FBS for expansion and 5% FBS when plated for experiments. Pilot experiments to determine the correct cell density to prevent overcrowding and standardize treatments across plate types were performed. All experiments were repeated on at least 3 separate occasions, representing separate experimental days at least one passage apart. All cells were from between passage numbers 39 and 45.

Approximately 8 h after plating (or 36 h for silencing experiments; see below), microglia were treated with PFT-µ (at doses indicated, diluted in DMSO to a final concentration of not more than 0.1%) or PBS (with 0.1% DMSO, ‘vehicle’, VEH). After 2 h, the cells were exposed to PBS (VEH; 1 µl/ml of PBS), LPS at 1 µg/ml [30,31] or IL-4 at 20 ng/ml [22,32]. After various exposure times (30, 60 and 120 min, 16 h) supernatant (conditioned media) was collected and stored at −80°C until analysis of cytokine/chemokine levels, and cells were harvested for Western blotting or RNA extracted for gene expression analysis.

### RNA Extraction and Quantification of Gene Expression by Real-Time qPCR

Total RNA from microglial cell cultures was extracted with the RNeasy mini kit according to the manufacturer's instructions (Qiagen, Courtaboeuf, France). RNA quality and concentration were assessed by spectrophotometry with the NanoDrop™ apparatus (Thermo Scientific, Wilmington, Del., USA). Total RNA (1-2 µg) was subjected to reverse transcription using the iScript™ cDNA synthesis kit (Bio-Rad, Marnes-la-Coquette, France). qPCR was performed in duplicate for each sample using SYBR Green Supermix (Bio-Rad) for 40 cycles with a 2-step program (5 s of denaturation at 96°C and 10 s of annealing at 60°C). Amplification specificity was assessed with a melting curve analysis. Primers were designed using Primer3 software. Sequences and their NCBI references are given in online supplementary table 1 (for all online suppl. material, see www.karger.com/doi/10.1159/000370031). The relative expression of genes of interest was determined relative to the expression of the reference gene, *GAPDH* (glyceraldehyde 3-phosphate dehydrogenase). Analyses were performed with the Bio-Rad CFX Manager 2.1 software.

### Multiplex Cytokine/Chemokine Assay

Microglia media harvested at different time points following treatment initiation was centrifuged briefly to remove particulates (300 *g* for 10 min). Cytokine and chemokine levels in the microglial media were measured using a Bio-Plex 200 with a 96-well magnetic plate assay according to the manufacturer's instructions (Bio-Rad). Cytokines and chemokines measured included IL-1α, IL-1β, IL-2, IL-6, IL-10, IL-12 (p70), IL-13, G-CSF, GM-CSF, IFNγ, TNFα, CXCL1 (KC), CCL2 (MCP-1), and CCL5 (RANTES). All samples were run in duplicate and data were analyzed with the Bio-Plex Manager software.

### Cell Viability (Mitochondrial Activity) Assay

Microglial viability was quantified using MTT [3-(4,5-dimethylthiazol-2-yl-)-2,5-diphenyl-2H-tetrazolium bromide; Sigma]. In this assay MTT, a tetrazolium dye, is bioreduced by the mitochondria into a formazan product that is insoluble in tissue culture medium [33]. In brief, MTT was added to a final concentration of 250 µg/ml to cells at various time points following treatment with PBS, LPS or IL-4 with or without PFT-µ. After 30 min, formazan was dissolved in DMSO and the absorbance was measured at 490 nm using a spectrophotometer (Glomax Multi+; Promega, UK).

### Phagocytosis Assay

Phagocytosis of fluorescently labelled *Escherichia coli* particles by BV2 was assessed using the Vybrant phagocytosis assay (V-6694; Invitrogen) according to the manufacturer's instructions. In brief, 50,000 cells were plated in 48-well plates and after 16 h of incubation with LPS with and without PFT-µ, the medium was changed to serum-free media containing the recommended suspension of bioparticles. Cells were incubated for 5 h before the particle-containing media was removed, washed twice with serum free media and incubated with a solution of trypan blue for 1 min to quench extracellular fluorescence and 1 ml of serum-containing media added to each well. The absorbance of each well (including cell-free, bead-free and media-free controls) was read. To adjust for cell density, following reading of the plate the cells were used in an MTT assay. The cells were also visually inspected for fluorescence and images were acquired on an EVOS® FL cell imaging microscope (Life Technologies, UK).

### Gene Silencing Experiments

Synthetic RNA duplexes for p53 (AM16708, *Trp53*; Life Technologies) and HSP-70 (SR418598, *Hspa1*; OriGene Technologies) or scrambled controls (all 100 nM) were transfected into BV2 cells using the INTERFERin reagent (Polyplus-transfection SA, Illkirch, France) in accordance with the manufacturer's protocol. Validation of silencing efficiency was made with Western blotting for the respective proteins. Cells were used in experiments 24 h after transfection.

### Western Blotting

Western blotting was performed as previously described [34]. The cells were homogenized directly in the wells, using RIPA buffer and a rubber policeman, followed by brief sonication (10× 1-second pulses of 20% max; Sonics Vibra-Cell VCX130; Sonics & Materials, Newtown, Conn., USA). Samples were resolved by 4-12% SDS PAGE (Invitrogen) and transferred to PVDF via a semi-dry blotting apparatus (Bio-Rad). Membranes were incubated with primary antibodies and the appropriate fluorescently tagged secondary antibody under the conditions listed in online supplementary table 2. Immunoreactive bands were visualized using the Odyssey Clx infrared scanner (Licor Biosciences), and analysis was performed on the Image Studio software package (Licor Biosciences).

### Nomenclature of Microglial Phenotype

We have adopted nomenclature consistent with a large body of research describing the phenotype of LPS- or IL-4-activated microglia, the classically activated M1-like phenotype and the alternately activated M2-like reparatory and regeneration-supporting phenotype [23,24,25,35,36].

### Statistics

Data are from three or more independent microglial cultures and are presented as means ± SEM. Data were assessed for normality. Gene and protein expression over time was analyzed with an ANOVA and a Bonferroni posttest. Statistics used for each data set and the significance of each are shown on the graphs and described within the figure legend. Analyses were performed with GraphPad 5.0 software (San Diego, Calif., USA), and p ≤ 0.05 was accepted as statistically significant.

## Results

### PFT-µ at Low Concentrations Does Not Effect Cell Health/Viability

**Fig. 1:**
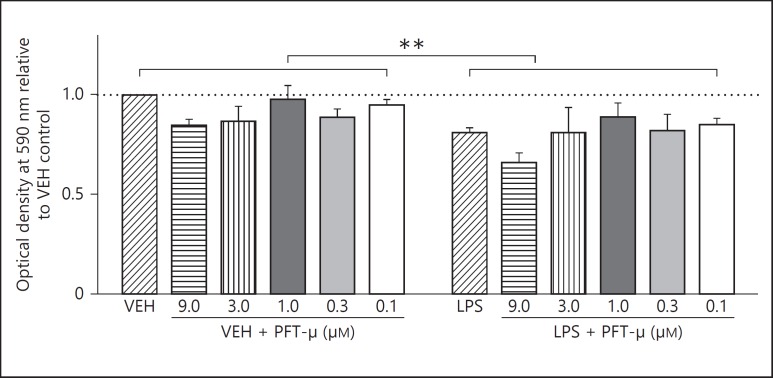
Effects of LPS and PFT-µ on cell health as assessed with MTT demonstrates that LPS alone reduced MTT output but there was no significant effect of PFT-µ. Mean ± SEM (n = 4). ** p < 0.01 (two-way ANOVA for effect of LPS).

To assess the effects of PFT-µ on BV2 health and viability to determine the appropriate doses for later testing we used doses between 0.01-9.0 µM with and without exposure to 1 µg/ml LPS. LPS reduced MTT output by approximately 15%, an effect previously described [37,38], and PFT-µ had no significant effect in the 2-way ANOVA (fig. 1). This observation was validated using a lactate dehydrogenase assay and cell estimates based on Hoechst staining (data not shown).

### In the Absence of LPS, Treatment with PFT-µ Reduces SOCS3 and p53 Gene Expression

**Fig. 2:**
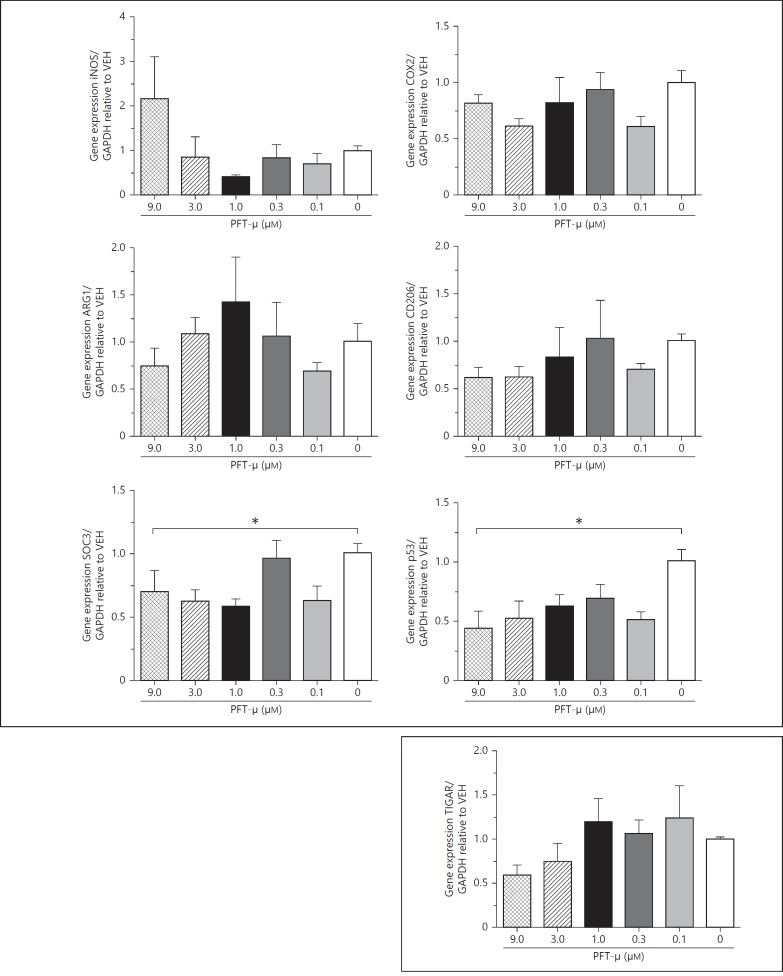
Gene expression relative to VEH. Exposure to PFT-µ in the absence of inflammatory stimulus reduces SOC3 expression and decreases p53 gene expression. Mean ± SEM (n = 4). * p < 0.05 (one-way ANOVA).

We initially used a set of phenotype markers to assess the effects of PFT-µ on the in vitro basal phenotype of BV2 microglia. Where basal-state BV2 were exposed to increasing doses of PFT-µ no change in mRNA expression was observed for inducible nitric oxide synthase (iNOS), cyclooxygenase-2 (COX2), arginase-1 (Arg1), and cluster of differentiation (CD) 206. However, there was a reduction in gene expression for the suppressor of cytokines-3 (SOCS3), a marker of an LPS-induced phenotype. PFT-µ also caused a significant reduction in gene expression for p53 but had no effect on TP53-inducible glycolysis and apoptosis regulator (TIGAR) expression (fig. 2).

### In the Presence of LPS, Treatment with PFT-µ Dose-Dependently Decreases the Acquisition of an LPS-Induced Phenotype

**Fig. 3:**
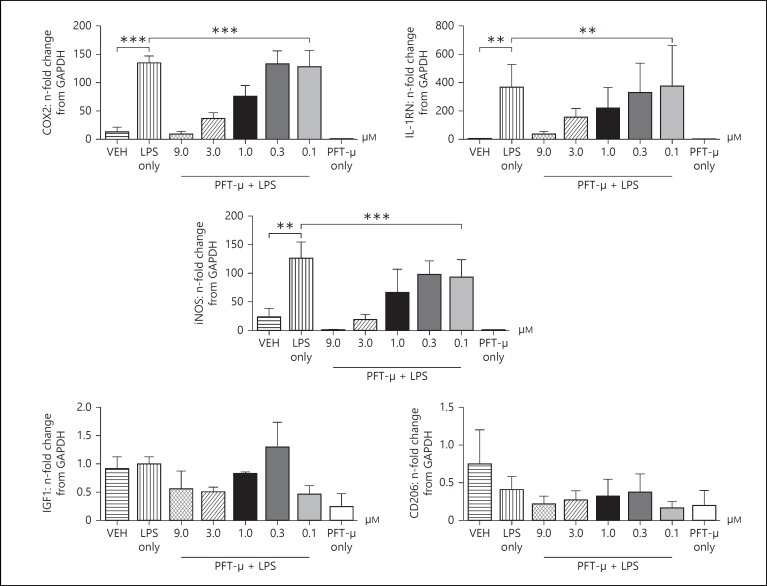
Gene expression: n-fold change from GAPDH. PFT-µ dose dependently reduces expression of M1-like genes induced in response to LPS. Mean ± SEM (n = 3/4). ** p < 0.01; *** p < 0.001 (one-way ANOVA).

**Fig. 4:**
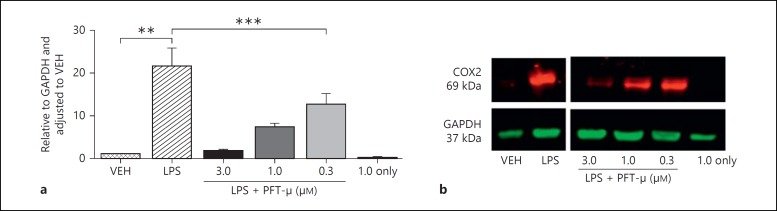
PFT-µ decreases the LPS-induced expression of COX2 protein in BV2 (quantified by Western blot). ** p < 0.01 (Student's t test); *** p < 0.001 (one-way ANOVA comparing LPS with and without PFT-µ).

Where BV2 were pretreated with PFT-µ 2 h prior to 1 µg/ml of LPS exposure a robust dose-dependent reduction was observed in the induction of typical M1/2b-like markers Ptgs2 (COX2), IL-1Rn and iNOS (fig. 3). Neither LPS nor PFT-µ had any significant effect on the gene expression of M2a markers Arg1 and CD206 (fig. 3) or IGF1 and Gal3 (data not shown). Where PFT-µ and LPS were coapplied there was a small but nonsignificant effect on the expression of phenotype markers (data not shown). As our previous experiments had identified significant alterations in mRNA expression, we went on to analyze BV2 protein lysates treated with PFT-µ. We chose to validate COX2 as its expression is robustly and consistently associated with LPS-induced activation [25] and perinatal brain injury [39,40]. We demonstrated a strong LPS-induced induction of protein expression and, conversely, a striking dose-dependent decrease in the presence of PFT-µ (fig. 4). Similarly, analysis of soluble factors in the media of BV2 exposed to LPS with and without PFT-µ revealed a dose-dependent reduction in LPS-induced chemokine/cytokine production by PFT-µ (table 1). PFT-µ had no effect at any dose trialed on the expression of genes (Arg1 and CD206) or protein (Arg1) induced by exposure of BV2 to 20 ng/ml of IL-4 to simulate an M2-like phenotype (data not shown).

### PFT-µ Has No Effects on the LPS-Induced Changes in Metabolism-Related Genes

LPS exposure caused reductions in gene expression for the metabolic markers PGC1α (peroxisome proliferator-activated receptor gamma coactivator 1-alpha; 198.70 ± 54.97 vs. 95.08 ± 5.54, p < 0.05, t test), NOXA (Latin word for damage; a proapoptotic member of the Bcl-2 protein family; 223.40 ± 75.10 vs. 104.7 ± 5.023, p = 0.067, t test) and TIGAR (6.67 ± 0.71 vs 1.16 ± 0.12, p = 0.061, t test). These changes were not recovered by exposure to PFT-µ (p > 0.05, one-way ANOVA).

### PFT-µ Has No Effects on the LPS-Induced Increases in Phagocytosis or Change in Morphology

**Fig. 5:**
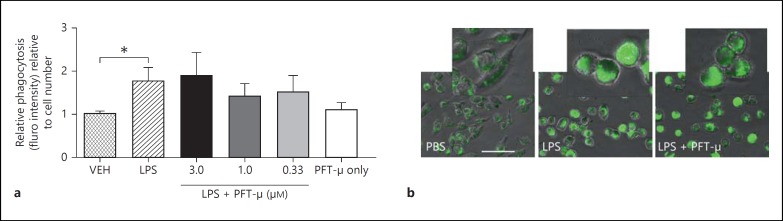
PFT-µ has no effect on the LPS-induced increase in phagocytosis (**a**) or the observed LPS-induced change in morphology (**b**). Mean ± SEM (n = 6). * p < 0.05 (Student's t test).

To assess any direct functional effect of PFT-µ exposure we treated BV2 with LPS with and without PFT-µ and assessed the uptake of fluorescently labeled *E. coli*. Activation with LPS caused a significant increase in phagocytosis, which was not altered by exposure to PFT-µ (fig. 5a). Qualitative assessment of morphology also revealed that PFT-µ did not prevent the LPS-induced change in BV2 morphology - a loss of processes and more refringent amoeboid appearance (fig. 5b).

### PFT-µ Decreases HSP-70 Protein Expression at a Late but Not Early Time Point

**Fig. 6:**
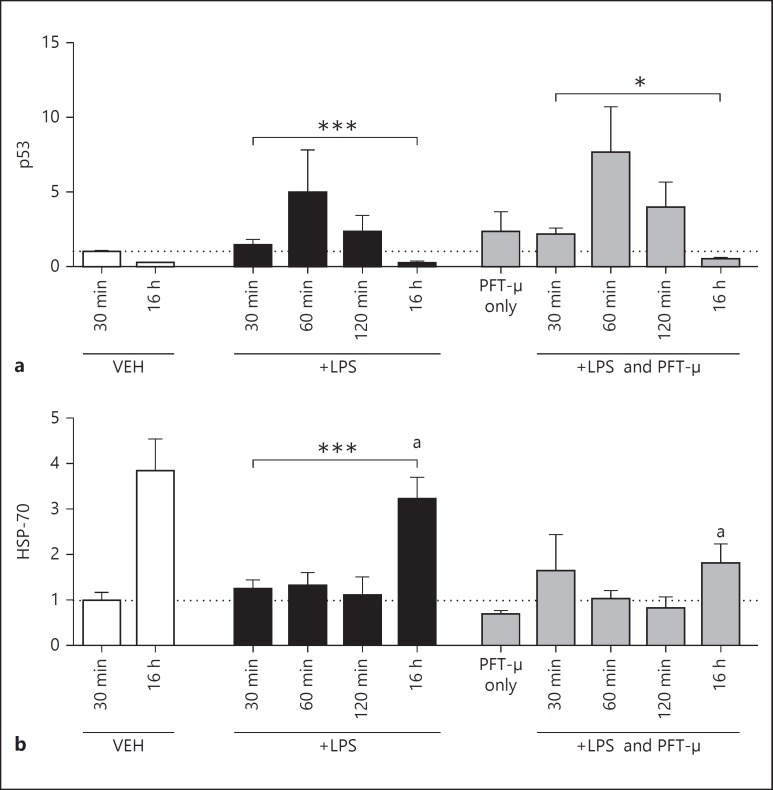
Protein expression relative to GAPDH and adjusted to VEH. PFT-µ effects on protein expression of p53 (**a**) and HSP-70 (**b**) in response to LPS. Mean ± SEM (n = 4/5). * p < 0.05; *** p < 0.001 (one-way ANOVA for effects over time); *a*: p < 0.05 (Student's t test).

Not only does PFT-µ modulate p53-mediated pathways, but it is also reported to alter HSP-70-mediated signaling [41]. Therefore, to assess the effects of LPS with and without PFT-µ on protein levels of both p53 and HSP-70 in BV2 cells, we performed Western blots on whole cell lysates at 30, 60 and 120 min and 16 h after exposure (fig. 6). We noted that PFT-µ was able to alter the significant change over time induced by LPS in HSP-70, and that this was due to a reduction in protein expression at 16 h by PFT-µ (fig. 6b). We also validated the well-characterized actions of PFT-µ - to have no effect on the LPS-induced nuclear translocation of p53 but reduce protein levels at the mitochondria (data not shown).

### Knockdown of HSP-70 Protein but Not p53 Protein Causes a Small Reduction in the Efficacy of PFT-µ

**Fig. 7:**
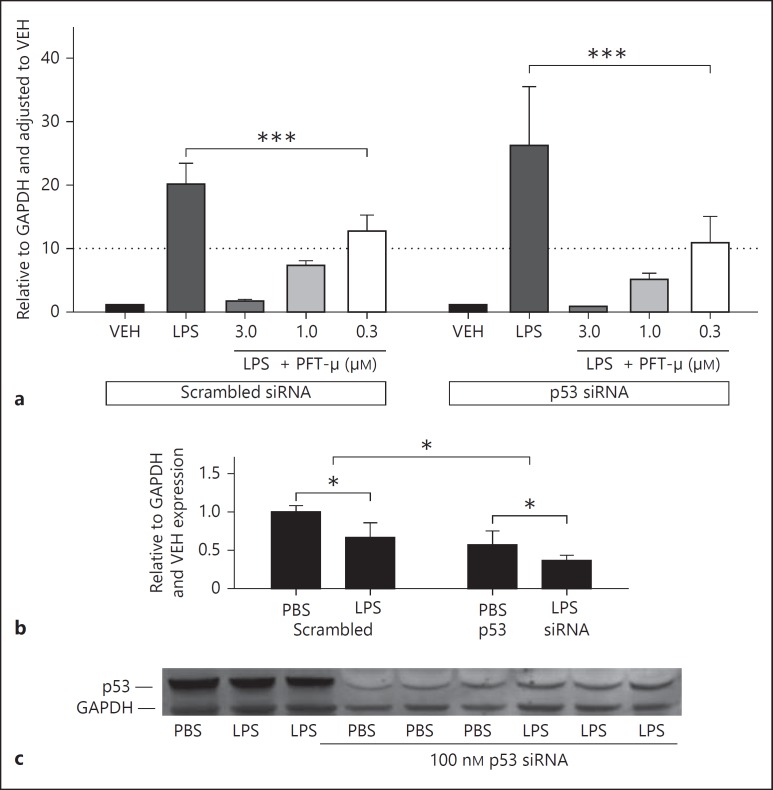
**a** Silencing of p53 expression has no effect on the how PFT-µ reduces LPS-induced COX2 protein expression (quantified by Western blot). **b** Validation of the effects of p53 siRNA on p53 protein expression. **c** A representative Western blot. Mean ± SEM (n = 5). * p < 0.05 (one-way ANOVA for effect of LPS and siRNA on expression of p53 protein); *** p < 0.001.

Genetic knockdown of p53 *(trp53)* with siRNA had no effect on the expression of COX2 protein by BV2 in response to VEH or LPS in the absence of PFT-µ (data not shown). The efficacy of PFT-µ in reducing the LPS-induced COX2 expression was not affected by silencing p53, and the same significant dose-dependent decrease in COX2 was observed (fig. 7a). Activation with LPS was associated with a decrease in p53 protein; this was reduced by approximately 50% with siRNA against p53 (fig. 7b, c).

**Fig. 8:**
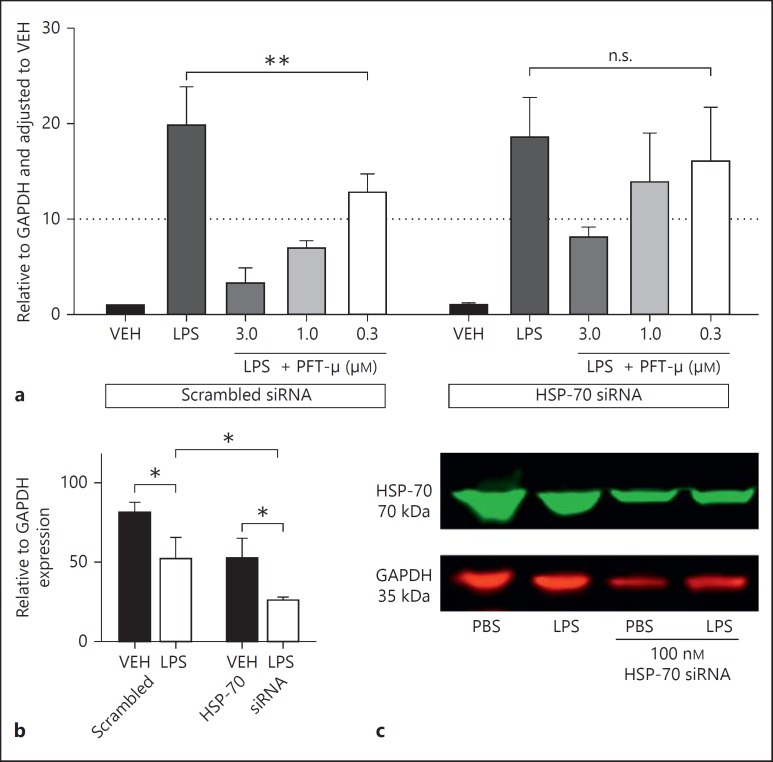
**a** Knockdown of HSP-70 gene expression decreases the ability of PFT-µ to reduce LPS-induced COX2 protein expression (quantified by Western blot). n.s. = Nonsignificant. **b** Validation of HSP-70 siRNA. **c** Representative Western blot. Mean ± SEM (n = 3/4). * p < 0.05 (two-way ANOVA for effect of LPS and siRNA on expression of HSP-70 protein); ** p < 0.05 (one-way ANOVA for effect of PFT-µ on COX2 expression).

Silencing HSP-70 (*Hspa1a*) gene expression decreased the efficiency of PFT-µ to reduce COX2 expression from LPS-stimulated BV2 (fig. 8a). We noted that the dose-dependent reduction in COX2 was not observed in HSP-70 siRNA-treated BV2. Activation with LPS was associated with a decrease in HSP-70 protein; this was reduced by approximately 50% with siRNA against HSP-70 (fig. 8b, c). Neither silencing of p53 nor HSP-70 had any significant effect on cell health as measured by MTT assay (data not shown).

## Discussion

**Fig. 9:**
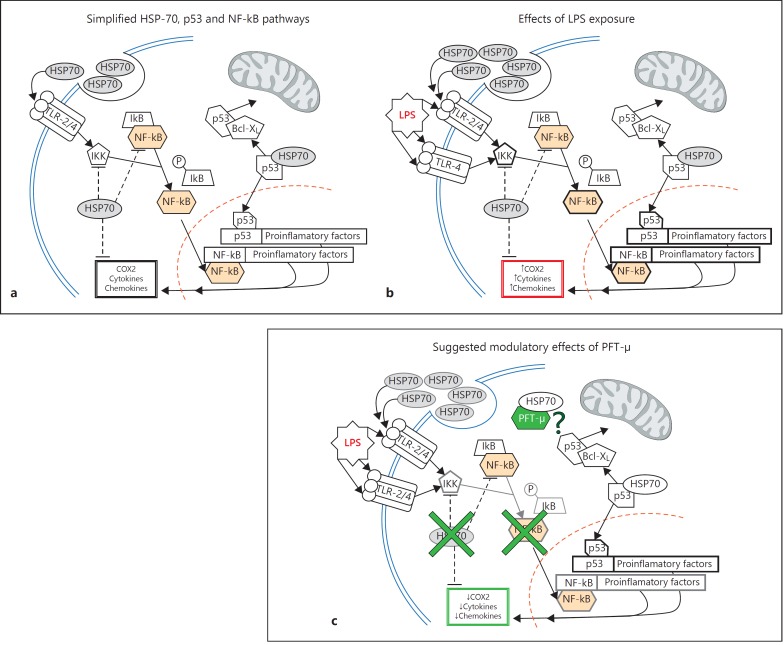
Simplified schematic representations of the HSP-70, p53 and NF-kB pathways (**a**) showing the known effects of LPS (**b**). **c** Proposed effects of PFT-µ, including a decrease in HSP-70 expression, decreased NF-kB pathway activation and an unknown effect of the HSP-70/PFT-µ complex (adapted from Hofacer et al. [66]).

This study suggests that a proportion of the neuroprotective effects reported for PFT-µ probably relates to a potent ability to reduce the M1-like proinflammatory activation of microglia. We observed that PFT-µ was able to reduce gene expression for multiple markers of an M1-like phenotype, protein expression of COX2 and the release of soluble inflammatory mediators. Interestingly, PFT-µ did not reduce the phagocytic ability of M1-like BV2. Genetic silencing experiments indicate that HSP-70 probably plays a role in mediating the effects of PFT-µ in reducing an M1-like phenotype in microglia (fig. 9).

### Are the Anti-Inflammatory Effects of PFT-µ Mediated via Mitochondrial p53 in Microglia?

PFT-µ inhibits p53 binding to the mitochondria and nonconditional p53 KO mice are known to have impaired mitochondrial respiration and increased glycolysis [13,42]. In addition, studies by Garden et al. [43] and Jayadev et al. [44] have demonstrated that the nuclear actions of p53 are involved in the acquisition of an M1-like phenotype - with a 30% reduction in the in vitro production of LPS-induced TNFα in nonconditional p53 KO microglia - but any specific role for mitochondrial p53 (separate from transcriptional changes) in activation is unknown. We observed that LPS exposure drove p53 to the nucleus and the mitochondria (data not shown) but decreased total cellular expression as expected [18,45], although the total cell levels were lower. Silencing of p53 in this study (≈50% decrease) did not influence the efficacy of PFT-µ. This would suggest that there is a limited role for mitochondrial p53 in the acquisition of microglial phenotype in this context (fig. 9). However, further targeted studies are needed to confirm this.

### What Is the Possible Role for HSP-70 in Mediating the Effects of PFT-µ in Microglia?

In addition to p53 we investigated the effects of HSP-70 silencing on the efficacy of PFT-µ based on strong evidence for actions via this pathway from transformed and normal cell studies [15,46]. The ≈50% reduction in HSP-70 protein expression was able to reduce the anti-inflammatory efficacy of PFT-µ. However, due to the partial effect of our observations, we speculate that the presence of a PFT-µ/HSP-70 dimer or the rapid appearance of liberated substrates also plays a role in the anti-inflammatory effects of PFT-µ. This is because removing HSP-70 only partly mimicked the effects of PFT-µ. Alternatively, the sustained loss of HSP-70 (due to the time taken to ensure silencing) may cause a compensatory shift in substrate expression or alternate pathways altering PFT-µ efficacy. Reducing the availability of HSP-70 leads to the loss of expression of binding partners, including EGFR, Erbb2 and Akt [15,47], which could have as yet unknown effects on PFT-µ functions.

Furthermore, altering the expression of HSP-70 is proposed to have two conflicting effects on inflammatory processes. Firstly, HSP-70 is released from myeloid cells stimulated with LPS [48], and this has proinflammatory effects by activating TLRs, leading to the production and release of factors such as TNFα [49,50]. Conversely, intracellular HSP-70 is anti-inflammatory due to its ability to sequester NF-kB and inhibit the activity of IKK, an enzyme responsible for NF-kB signaling pathway activation [51,52]. The NF-kB pathway is crucial in the inflammatory and cell stress response [53]. As such, although we can infer that PFT-µ requires the inhibition of HSP-70 substrate binding for its anti-inflammatory functions, the nature of this remains obscure.

In addition to preventing the substrate binding of HSP-70, PFT-µ has a strong antagonistic effect on canonical NF-kB signaling, reducing basal and TNFα-induced pathway activation via stabilization of the inhibitory protein IKBa [41] and inhibiting nuclear translocation and phosphorylation [46]. The release of chemokines and cytokines in response to LPS from microglia involves the activation of the canonical NF-kB pathway [for reviews, see [54,55]]. It has been reported that, in vivo, this action of PFT-µ causes a decreased inflammatory response to LPS characterized by decreased production of iNOS, TNFα and IL-6 from the liver [46]. The dose-dependent reduction in the release of chemokines and cytokines in our study together with the lack of efficacy of the two alternate pathways of action for PFT-µ strongly suggests this pathway is being targeted by PFT-µ (fig. 9).

### Can We Infer that PFT-µ Plays a Protective Role in Experimental Brain Injury via Actions on Microglia?

PFT-µ has previously been shown to provide striking protection in a mouse model of HI-induced NE [12]. Interpretation of this previous work focused on the role of p53 in neurons, and it is known that PFT-µ strongly inhibits the stress-induced translocation of p53 to the mitochondria, preventing mitochondrial outer membrane permeabilization and cell death [12,13]. However, in addition to cell death the release of pro-inflammatory cytokines and activation of microglia play pathological roles in this model [56,57,58]. As such, our data, showing reduced M1-like microglial activation and release of soluble proinflammatory factors, support our hypothesis that direct effects of PFT-µ on microglia may contribute it its neuroprotective role. Although our study presents robust support for a microglia-specific facet to PFT-µ-mediated neuroprotection, we note that BV2 cells do not have an identical transcriptomic profile to ex vivo or primary microglia [59]. However, in response to LPS, around 90% of genes that were regulated in primary microglia were also reported to be regulated in BV2 [28]. It has been reported that PFT-µ treatment reduces LPS-induced liver inflammation [46], suggesting these effects would also be observed in vivo. The following further support our hypothesis: (1) a NF-kB antagonist (tat-NBD) had very similar neuroprotective effects to those of PFT-µ [17] and (2) using IL-1 receptor blockade or strategic anti-inflammatory strategies also reduces injury in this model [60,61].

A further potential contributor to the protective role of PFT-µ is that it maintained the activation-induced levels of phagocytosis while having robust anti-inflammatory effects. Phagocytosis is important in repair and regeneration after injury in the perinatal and adult central nervous system, which is demonstrated by an exacerbation of injury following its inhibition [21,62,63]. Exposure of BV2/microglia to LPS or alternate M1-like phenotype inducers has previously been reported to increase phagocytosis in line with our observations [64,65].

In conclusion, PFT-µ robustly inhibits the LPS-induced M1-like activation of BV2 microglia but has no effects on LPS-induced phagocytosis. These data suggest that the strong neuroprotective effects reported for PFT-µ in a model of perinatal HI-induced NE include modulating inflammation and repair. We suggest that the actions of PFT-µ on microglia are most likely linked to HSP-70 and to a great extent NF-kB. This study strengthens the justification for additional trials of PFT-µ across models of perinatal brain injury to fully assess its clinical applicability.

## Figures and Tables

**Table 1:** Protein levels in media from BV2 treated with LPS with and without PFT-μ

Cytokine/chemokine	LPS as n-fold change from VEH	p1	Change from VEH				p2
			LPS + 9.0 μM PFT-μ	LPS + 3.0 μM PFT-μ	LPS + 1.0 μM PFT-μ	LPS + 0.3 μM PFT-μ	
IL-1ß	1.73±0.17	<0.05	0.93±0.33	1.30±0.36	1.58±0.12	1.82±0.38	<0.08
IL-4	1.69±0.18	<0.05	0.96±0.27	1.36±0.31	1.53±0.1	1.76±0.33	
IL-6	60.00±22.64	<0.08	2.85±2.26	27.45±15.19	45.75±23.03	69.45±21.37	<0.08
IL-10	1.53±0.15	<0.05	0.98±0.17	1.27±0.22	1.40±0.18	1.61±0.24	
IL-12	1.85±0.24	<0.05	1.01±0.23	1.44±0.32	1.63±0.20	1.94±0.37	
IFN-γ	1.82±0.23	<0.05	0.92±0.27	1.35±0.34	1.60±0.18	1.89±0.39	<0.08
KC	4.29±2.16	<0.05	1.05±0.31	3.74±1.33	4.67±1.36	5.81±2.32	
MCP-1	42.86±16.93	<0.05	1.18±0.77	8.79±4.67	25.71±16.28	39.75±17.19	<0.08
MIP-1	1.60±0.11	<0.05	0.77±0.38	1.18±0.38	1.48±0.03	1.61±0.39	<0.08
TNF-α	5.67±0.69	<0.05	1.05±0.15	2.08±0.53	2.76±2.39	6.06±0.71	<0.08

Values are means ± SEM (n = 4). p1: compared to VEH (t test). p2: effect of PFT-μ (one-way ANOVA).
